# A Case Report of Pseudotumor Cerebri Syndrome with a Huge Retroperitoneal Cyst

**DOI:** 10.3390/medicina59091573

**Published:** 2023-08-29

**Authors:** Jae-Myung Kim, Hyunsoo Kim, Kyung Wook Kang, Seong-Min Choi, Man-Seok Park

**Affiliations:** 1 Department of Neurology, Chonnam National University Hospital, Chonnam National University Medical School, Gwangju 61469, Republic of Korea; etj3060@hanmail.net (J.-M.K.); kharistma@naver.com (H.K.); kang8774@hanmail.net (K.W.K.); 2 National Research Center for Dementia, Gwangju 61469, Republic of Korea

**Keywords:** intracranial pressure, pseudotumor cerebri, headache, papilledema

## Abstract

*Background*: Aside from primary pseudotumor cerebri syndrome (PTCS) with an unknown etiology (i.e., idiopathic intracranial hypertension), which typically occurs in association with obesity, several conditions including cerebral venous abnormalities, drug use, and hormonal imbalance may be a secondary cause of PTCS. However, a focal space-occupying lesion outside of the brain as a cause of PTCS has rarely been reported. *Case Presentation*: A previously healthy 34-year-old man presented with blurred vision for three weeks. The patient had a three-month preceding history of worsening headache. On admission, he was hypertensive (160/90 mmHg) and underweight with a body mass index of 18.4 kg/m^2^. Fundus examination documented papilledema in both eyes. Neurological examination was unremarkable except for mild nuchal rigidity, and results of routine serologic testing were normal. Gadolinium-enhanced brain magnetic resonance imaging revealed bilateral posterior scleral flattening, suggesting intracranial hypertension. There was no other abnormal brain parenchymal lesion or meningeal enhancement. Cerebrospinal fluid (CSF) assay showed a markedly increased opening pressure (30.0 cmH_2_O) with normal CSF composition. A tentative diagnosis of PTCS was made based on ophthalmological, neuroradiological, and laboratory findings. During differential diagnosis, abdomen computed tomography demonstrated a huge benign cystic lesion (14.7 × 10.6 × 16.4 cm) in the right retroperitoneal space, which originated from the mesentery and resulted in hydronephrosis and renovascular hypertension due to external compression of the right kidney. Other evaluations were unremarkable. After successful surgical removal of the cyst, clinical symptoms such as headache, blurred vision, and papilledema on fundus examination were markedly improved, and blood pressure was normalized during the three-month follow-up period. *Conclusions*: A large retroperitoneal cyst that can increase intra-abdominal pressure could be a rare cause of PTCS. Therefore, meticulous evaluation is warranted for patients with PTCS, especially those without known risk factors.

## 1. Introduction

Pseudotumor cerebri syndrome (PTCS) is characterized by increased intracranial pressure (ICP) with papilledema, normal brain parenchyma on neuroimaging, and normal cerebrospinal fluid (CSF) composition [1]. Aside from primary PTCS with an unknown etiology (i.e., idiopathic intracranial hypertension), which typically occurs in association with obesity or recent weight gain, several conditions including cerebral venous abnormalities, drug use, and hormonal imbalance may be a secondary cause of PTCS [1]. Specifically, a focal space-occupying lesion outside of the brain as a cause of PTCS has rarely been reported [1]. Here, we report a case of PTCS with papilledema in a patient who was successfully treated with surgical removal of a huge retroperitoneal cyst and suggest a possible hypothetical mechanism. This case report was prepared following the CARE Guidelines [2].

## 2. Case Presentation

**Figure 1:**
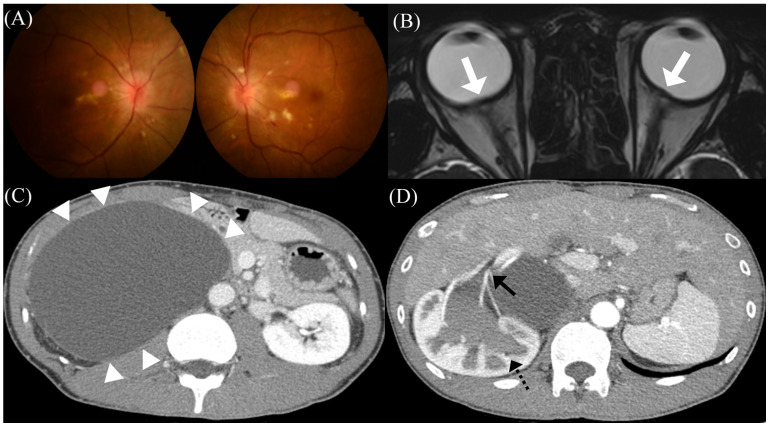
Initial clinical and radiological presentations of the patient. (**A**) Initial fundus photography showing bilateral papilledema and visible hard exudates at the posterior pole. (**B**) T2-weighted brain magnetic resonance images showing the flattening of the posterior sclera bilaterally, suggestive of intracranial hypertension (white arrows). (**C**) Abdominal computed tomography with contrast enhancement showing a huge benign cystic lesion (white arrowheads), (**D**) resulting in hydronephrosis (black dotted arrow) and (**D**) extrinsic compression of the renal artery of the right kidney (black arrow).

A previously healthy 34-year-old man presented with blurred vision for three weeks. The patient had a three-month preceding history of worsening headache that was moderate to severe in intensity, occurred in the whole cranium, was pulsatile in nature, and was accompanied by nausea/vomiting. He reported that conventional medications for migraine were ineffective. On admission, he was hypertensive (160/90 mmHg); however, heart rate and body temperature were normal. He was underweight with a body mass index of 18.4 kg/m^2^. Fundus examination revealed papilledema in both eyes (Figure 1A). His best-corrected visual acuity was 20/20 in the left eye and 20/22.5 in the right eye. Pupillary light reflex was normal, and visual field defect was absent. Neurological examination was unremarkable except for mild nuchal rigidity. The results of routine serologic testing, including complete blood count, markers of inflammation (e.g., C-reactive protein, erythrocyte sedimentation rate), electrolytes, and blood chemistry, were all normal. Gadolinium-enhanced brain magnetic resonance (MR) imaging revealed bilateral posterior scleral flattening, suggesting intracranial hypertension (Figure 1B). However, there was no other abnormal brain parenchymal lesion or meningeal enhancement. CSF assay showed a markedly increased opening pressure (30.0 cmH_2_O) without pleocytosis. In addition, the protein concentration and serum/CSF glucose ratio were all within the normal range.

Although a tentative diagnosis of PTCS was made based on ophthalmological, neuroradiological, and laboratory findings, he had neither any common risk factors for primary PTCS (e.g., obesity, recent weight gain) nor a history of drug exposure/medical illness. Therefore, we performed more extensive work-ups for the secondary cause of PTCS, including hormonal studies, brain MR venography, chest/abdomen computed tomography (CT), autoimmune assessment, and tumor marker detection. Although the patient did not show any abdominal symptoms or signs (e.g., pain, distention), abdomen CT with contrast enhancement revealed a huge benign cystic lesion (14.7 × 10.6 × 16.4 cm) in the right retroperitoneal space, which originated from the mesentery and resulted in hydronephrosis due to external compression of the right kidney (Figure 1C,D). The lesion was pathologically confirmed to be a mesenteric benign cyst. There were neither evidence for mechanical compression nor hormonal abnormalities [e.g., adrenocorticotrophic hormone (ACTH) stimulation test, aldosterone-to-renin ratio] suggestive of adrenal gland involvement. Captopril renal scintigraphy confirmed that his high blood pressure was associated with renovascular hypertension of the right kidney. Other evaluations for the secondary cause of PTCS were unremarkable. Transthoracic echocardiography showed mild left ventricular hypertrophy with a normal ejection fraction of 60.3%, without other chamber or valvular abnormalities. Spirometry demonstrated a normal forced expiratory volume in 1 s/forced vital capacity (FEV_1_/FVC = 98.1% predicted) with a decreased FVC (73% predicted), which indicated a restrictive pattern.

**Figure 2:**
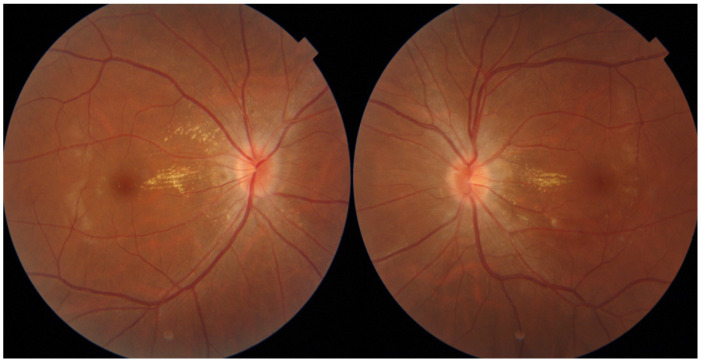
Follow-up fundus photography of the patient. Follow-up fundus photography showing improved state of papilledema at three months after surgical removal of the cyst.

As underlying cardiopulmonary diseases or other potential causes of PTCS were not observed except for a large mesenteric cyst, surgical removal of the cyst was performed. Then, clinical symptoms such as headache, blurred vision, and papilledema on fundus examination were markedly improved, and blood pressure was normalized during the three-month follow-up period (Figure 2).

## 3. Discussion

The diagnosis of PTCS is often a clinical challenge since the common presenting symptoms of PTCS (e.g., headache, blurred vision), as observed in our case, are highly variable and easily overlooked as non-specific complaints [1,3]. Furthermore, as the incidence of PTCS is much lower in East Asia than in Western countries because severely obese patients with PTCS are rare, clinicians need to carefully consider risk factors other than obesity [4,5]. However, regardless of the etiology, without early recognition and prompt management, it may lead to devastating complications such as visual loss [3].

ICP may be raised in association with any changes resulting in increased venous sinus pressure, decreased CSF drainage, and enhanced CSF production [3]. Among them, obesity is a well-known precipitating factor of primary PTCS (up to 60–70%), and previous studies have demonstrated weight loss as an effective treatment option for obese patients with PTCS [3]. A worldwide incidence of PTCS is around 12–20 per 100,000 people per year in women who are obese and of childbearing age, whereas 0.5–2 per 100,000 people per year in the general population [3]. To explain the relationship between obesity and PTCS, several hypotheses have been suggested [3]. In particular, central obesity, possibly attributed to the physical effect of abdominal mass, may increase intra-abdominal pressure (IAP), resulting in pleural and cardiac filling pressure elevation and impeded venous return from the brain [1,3]. Furthermore, it may lead to increased intracranial venous pressure and reduced CSF absorption, which are associated with PTCS [1].

Similarly, the mechanical effects of a large space-occupying lesion in the abdominal cavity may cause a chronic increase in IAP even in the absence of other contributing factors [6]. Acute increases in IAP can lead to abdominal compartment syndrome characterized by multiple organ dysfunction, whereas chronic increases in IAP will predominantly affect the cardiorespiratory system [6]. Furthermore, previous studies showed that renal disease could be a secondary cause of PTCS; however, the proposed mechanisms included hormonal/electrolyte imbalance, uremia, or renal tubular necrosis rather than mechanical obstruction [7], which were all absent in the present case despite extrinsic renal compression caused by the cyst. In our case, the results of extensive work-ups for the differential diagnosis of PTCS were all unremarkable except for a huge retroperitoneal cyst. Moreover, surgical removal of the cyst improved both symptoms and signs associated with PTCS. Therefore, although there is still the possibility of an unknown etiology or coincidence, we hypothesize that increased IAP due to the mass effect of the cyst may have contributed to the development of PTCS in our case. Considering that the patient did not have known cardiopulmonary diseases or related symptoms, the following findings might also support our hypothesis: (1) Left ventricular hypertrophy documented on transthoracic echocardiography could be associated with renovascular hypertension or elevated cardiac filling pressure caused by increased IAP [8,9]. (2) A restrictive pattern on spirometry may be observed in association with various extrapulmonary conditions including increased intrathoracic pressure (which is possibly attributed to increased IAP) and pulmonary parenchymal disease [8].

In a literature review, there were several reports of spinal tumor, a space-occupying lesion outside of the brain, as a possible cause of secondary PTCS [10]. The authors suggested that cervical tumors which obstruct the upper spinal canal might lead to increased venous pressure and subsequent PTCS, whereas the pathophysiology in lower spinal tumors could be more complicated [10]. In detail, spinal obstruction could compromise the lumbosacral reservoir for CSF flow, impacting CSF compensation due to pressure alterations [10]. Otherwise, increased CSF protein might affect the elevation of CSF fibrinogen and CSF viscosity, resulting in reduced CSF absorption [10].

Increased ICP in PTCS may present various visual morbidities such as visual field loss or decreased visual acuity, which requires particular attention [3]. A previous study suggested that 29.1% of patients had reduced baseline visual acuity despite mild visual field loss [3]. Another study showed that decreased visual acuity was found in 22% of patients at diagnosis and 18% at final follow-up [3]. Moreover, papilledema, a diagnostic hallmark of PTCS, can be unilateral in up to 10% of patients with primary PTCS, evident in the late stage of the disease, or even absent in some cases [3]. Therefore, like other potential causes of increased ICP such as traumatic brain injury, ICP in PTCS should be monitored carefully to prevent permanent sequelae through non-invasive techniques including optical coherence tomography, ocular ultrasound, as well as observation of relevant clinical signs (e.g., cranial nerve disorder) [3,11].

In conclusion, a large retroperitoneal cyst that can increase IAP could be a rare cause of PTCS. Therefore, meticulous evaluation is warranted for patients with PTCS, especially those without known risk factors.

## Data Availability

The original contributions presented in this study are included in the article and further inquiries can be directed to the corresponding author.
